# The dual effect of background music on creativity: perspectives of music preference and cognitive interference

**DOI:** 10.3389/fpsyg.2023.1247133

**Published:** 2023-10-05

**Authors:** Xinyao Xiao, Junying Tan, Xiaolin Liu, Maoping Zheng

**Affiliations:** ^1^China Institute of Music Mental Health, Chongqing, China; ^2^School of Music, Southwest University, Chongqing, China; ^3^Guizhou University of Finance and Economics, Guiyang, China; ^4^School of Psychology, Southwest University, Chongqing, China

**Keywords:** background music, creativity, dual effect, emotional valence, cognitive interference, music preference

## Abstract

Music, an influential environmental factor, significantly shapes cognitive processing and everyday experiences, thus rendering its effects on creativity a dynamic topic within the field of cognitive science. However, debates continue about whether music bolsters, obstructs, or exerts a dual influence on individual creativity. Among the points of contention is the impact of contrasting musical emotions–both positive and negative–on creative tasks. In this study, we focused on traditional Chinese music, drawn from a culture known for its ‘preference for sadness,’ as our selected emotional stimulus and background music. This choice, underrepresented in previous research, was based on its uniqueness. We examined the effects of differing music genres (including vocal and instrumental), each characterized by a distinct emotional valence (positive or negative), on performance in the Alternative Uses Task (AUT). To conduct this study, we utilized an affective arousal paradigm, with a quiet background serving as a neutral control setting. A total of 114 participants were randomly assigned to three distinct groups after completing a music preference questionnaire: instrumental, vocal, and silent. Our findings showed that when compared to a quiet environment, both instrumental and vocal music as background stimuli significantly affected AUT performance. Notably, music with a negative emotional charge bolstered individual originality in creative performance. These results lend support to the dual role of background music in creativity, with instrumental music appearing to enhance creativity through factors such as emotional arousal, cognitive interference, music preference, and psychological restoration. This study challenges conventional understanding that only positive background music boosts creativity and provides empirical validation for the two-path model (positive and negative) of emotional influence on creativity.

## Introduction

Creativity, a seminal force behind scientific, technological, and cultural progression, is an indispensable facet of cognitive capability. Recognized as an essential trait in innovative individuals, it is manifested in heightened levels of curiosity, imagination, risk-taking endeavors, and penchant for challenge (Williams, 1979; Williams, 1993). Fundamental to this creative spirit are originality of thought and cognitive flexibility–elements identified by neuroscience as crucial constituents of creativity (Benedek et al., 2014). In addition, the fluency of ideation, a concept intrinsically linked to cognitive flexibility, is proposed to be fundamental to creativity (Guilford, 1967; Torrance et al., 1970). Innovative ideas or actions precipitate critical changes within a field or even establish novel domains. As defined in seminal psychological perspectives, creativity implies the genesis of original and socially applicable ideas, insights, or solutions to problems (Sternberg and Lubart, 1996; Fink et al., 2010). Creative individuals, harnessing the symbols inherent to their domains, come up with distinctive ideas and discern new patterns (Csikszentmihalyi, 1997). These individuals’ capacity to imbue novelty and usefulness into their concepts sets them apart and facilitates transformative change across various spheres of society.

Creativity is a multifaceted construct characterized by complexity and dynamism, pivoting around individual variances (Cropley and Cropley, 2008) and a myriad of environmental inputs (Russ, 1993; Vosburg and Kaufmann, 1999; Fernández-Abascal and Díaz, 2013). Intriguingly, the configuration of creativity is shaped in part by intra-individual factors, such as personality, personal predilections, and intellectual capacities. This attribute, in tandem with the copious situational stimuli encountered in our multifarious world, engenders a distinctive blend of external environmental influences, ranging from diverse cognitive stimuli–examples of which include brainstorming techniques and idea exchanges–to complex sociocultural dynamics (Paulus and Nijstad, 2003; Dugosh and Paulus, 2005). Recognizing the profound linkage between creativity and the above factors, it becomes apparent that comprehensive exploration of environmental catalysts, which could potentially enhance creativity in accordance with the idiosyncrasies of individuals, is both an intriguing scientific endeavor and a strategic move for future societal development. The intersection of this exploration with human psychology, scientific advancement, technological innovation, and the educational landscape has untapped implications (Merter, 2017; Andonova et al., 2023). Consider, for instance, music–an environmental factor with irrefutable influence on cognitive processing. Music possesses the capacity to bolster cognitive functionalities inclusive of memory, attention, and emotional processing (Brattico et al., 2011; O’Hare, 2011). The intentional incorporation of emotion-evoking music or music as an ambient background correlates esthetically with individual creativity, especially when it comes to spatial capabilities (Schellenberg, 2004; Schellenberg et al., 2007; Wang et al., 2020). Considering each individual’s unique psychological trajectory and musical preference, the interrelationship between music, emotion, and cognitive processing is part of a larger, more comprehensive tapestry. Understanding this tapestry may hold the key to further deciphering the dynamic construct that is creativity.

Music has been firmly recognized as a critical component influencing both emotional and cognitive processing (Chabris, 1999; Schellenberg, 2005). Over time, research investigating the induction of individual creativity through music has exhibited numerous rewarding insights. Notwithstanding this progress, an array of inconsistent findings has been reported, with the most contentious revolving around two key areas: the emotional valence of music and the context in which it is consumed (Nantais and Schellenberg, 1999; Thompson et al., 2001; Husain et al., 2002). While the former controversy engages deeply with the emotional valence of music, the latter pertains to the mobilization of cognitive resources. Recognized as an integral dimension of human emotion, emotional valence, also known as emotional pleasantness, is conventionally classified into positive valence - encapsulating emotions such as happiness, pleasure, excitement–and negative valence, incorporating feelings of sadness, pain, and anger. These classifications pivot around degrees of emotional pleasantness, wherein both happiness, characterized by higher emotional pleasantness, and sadness, marked by lower emotional pleasantness, have notable associations with creativity. The relationship between emotional valence and creativity has sparked rigorous academic debate, particularly around whether it is positivity, negativity, or a combination of both, that stimulates creativity. Naturally, this debate traverses into the realm of music and its interaction with emotional valence and creativity. Significant disparities are apparent across two genres of music profoundly intertwined with cognitive and emotional processing: instrumental music - categorically ‘pure’ - and vocal music, which is not bound by the lack of human voice. The disparate capacities of these music genres bear observable influence on cognitive processes like memory and attention, leading to plausible conjectures of likely variance in their effects on creativity when used as background music. Explicating these relationships raises two pertinent questions: Does music’s emotional valence exert a dual-pathway effect on creativity? Further, are there discernible differences between instrumental and vocal music’s influence on creativity when employed as background music?

### Background music and creativity

Background music is typically conceptualized as an auditory stimulus that is purposefully integrated into an environment. It is not intended for active listening, but rather to subtly modulate an individual’s mood, behavior, or perception of their surroundings (Radocy and Boyle, 2012). It harbors substantial impact on both emotional and cognitive processes, proving itself to be more than just an auditory accent in our daily lives. The theory of “emotional contagion” proposes that individuals subconsciously assimilate the emotional essence portrayed forth in music, engendering a powerful effect on their emotional state (Juslin and Västfjäll, 2008). Additionally, background music holds a sway over an array of cognitive processes, such as memory recall, attention, and most notably, creativity. Music, contingent upon the level of arousal it incites and the mood it evokes within listeners, can bring about either an enhancement or reduction in cognitive performance (Thompson et al., 2001). The presence of various stimuli within the music itself, such as the tempo, volume, structural complexity, and listener’s familiarity can produce varying effects. These music-induced stimuli can extend either facilitative or hindering influences on cognitive performance. Thus, apart from being a mere tool of esthetic enjoyment, music could potentiate, or possibly contravene, cognitive excursions into creativity.

Music, as an ambient factor, has been indirectly assessed for its influence on creativity, primarily within emotional valence and arousal parameters. Studies suggest that background music, modulating our mood, can either augment or diminish our cognitive faculties, contingent on the listener’s physiological arousal. Of note, music with moderate emotional arousal tends to boost cognitive functioning, primarily by establishing an optimal mental activation level and fostering greater fluidity and flexibility in thinking (Mehta et al., 2012; Threadgold, 2017). Certain prior investigations have ventured into the territory of evaluating how different emotional intensities of music, used as background conditions, exert influence on creativity. Preliminary findings indicate that background music does enhance creative capacities and that music embedded with different emotional cues yields differential effects, with positive mood music offering superior ameliorative results (Ritter and Ferguson, 2017). The reasoning provided for these observations rests on the proposal that the positive emotions, elicited through such music, can escalate arousal levels, transiently modulate an individual’s mood to be more positive, foster greater fluidity of thought and, consequentially, improve cognitive processing. This optimized cognitive processing could, in turn, generate more creativity (Basch, 1996; Fredrickson, 2001; Fredrickson and Branigan, 2005).

The fundamental theoretical framework supporting these assertions is the emotional arousal theory. It predicates that the facilitative effect of background music on creativity arises from its capacity to serve as an auditory stimulus that can transiently modulate emotional states. This modulation can activate the mind and enhance both the efficiency of memory retrieval and thought flexibility, thus augmenting the individual’s capacity to generate more creative solutions or notions (Fredrickson, 2003). This transient induction of positive emotions and escalated arousal can enhance cognitive flexibility (Thompson et al., 2001; Mehta et al., 2012). The relationship between musical arousal and cognitive processing functions along an inverted U-shaped curve, with cognitive processing being optimal at moderate levels of music-induced arousal. Below-optimal or excessively high arousal levels induced by background music may lead to subpar cognitive processing. This study underscores the intriguing interplay among music, mood, arousal, and creative cognition, while substantiating the hypothesis that music, particularly with different emotional inclinations, can be subtly employed to ignite the spark of creativity.

Drawing upon Cognitive Interference Theory, it is posited that background music acts as a hindrance to creativity. When participants undertake a creative task, auditory stimuli potentially distract cognitive resources. An overload of cognitive information can infringe on an individual’s cognitive processing capabilities, consequently diminishing emotional pleasure. This interference is likely to stifle creativity and adversely impact performance in cognitive processing tasks (Shek and Schubert, 2009). In simpler terms, a diverse range of auditory music presentations encompassing various pitches, timbres, rhythms, and tempos can deter creativity by diverting cognitive resources when individuals are engaged in cognitive processing tasks. Implicit in this theoretical perspective is the consideration that music is not always an aide in all situations; its influence might be situational or task-dependent. Thus, it sheds light on a deeper understanding of the intertwined complexities of music, emotion, and cognition.

Especially notable are the contrasting effects of instrumental music and vocal music on emotional and cognitive processing. Research indicates that creativity tends to align more closely with the presence of instrumental background music (Ritter and Ferguson, 2017). This is supported by observations that emotional arousal elicited by such instrumental stimuli moderately boosts participants’ cognitive flexibility and activates cognitive processing. Additionally, instrumental music does not disproportionately occupy attention or cognitive resources, thus avoiding cognitive disruption. In contrast, listeners subjected to vocal music often experience an over occupation of attention and cognitive resources, which can invariably disrupt cognitive functioning and inhibit creativity. However, some findings suggest that background music–whether instrumental or vocal – may impair cognitive processing when compared to conditions of silence. Consequently, neither type of music appears to facilitate creativity markedly better than a quiet background (Shek and Schubert, 2009; Threadgold et al., 2019). Considering these findings, it is crucial to navigate the complexity of music’s dual nature as both an emotional stimulant and a potential cognitive distraction. This balance suggests promising avenues for further research to better understand the nuanced relationship between music, emotional and cognitive processing, and creativity.

Contrary to this, alternative perspectives propose that instrumental music promotes cognitive processing by enhancing positive emotions and attenuating negative emotions, while vocal music impedes cognitive processing by augmenting negative emotions and reducing positive ones (Stratton and Zalanowski, 1994; Ali and Peynircioğlu, 2006; Mori and Iwanaga, 2014). Moreover, beyond “acoustic interference,” the concept of “semantic interference” has gained traction, underscoring the role of background music in hampering creativity. Songs are rife with linguistic elements, prompting the individual’s cognitive resources to be allocated for controlled semantic processing like refinement and language production, in addition to musical processing (Carretti et al., 2009; Marsh, 2009). When engaged in creative tasks, this dual processing demand potentially leads to a more pronounced cognitive conflict, thereby compromising performance. It’s pertinent to explore whether differing genres of background music significantly alter creativity levels, with potential implications in both academic and industrial contexts.

Extant research traverses various dimensions of creativity, bringing forward some tangible evidence that underpins the theories discussed earlier. In an exploration of how instrumental music influences creativity, a study utilized an “Alternative Use Task” (Guilford’s Alternative Uses Task, a classic test to measure creative thinking aspect of creativity) and revealed that music characterized by high emotional valence and high arousal (typically associated with happiness) could enhance individual creativity. Notably, it also seemed to bolster persistence and flexibility during the execution of a creative task when juxtaposed with a quiet condition or background music reflecting high emotional valence + low arousal (calm), low emotional valence + high arousal (tense), and low emotional valence + low arousal (sad) (Ritter and Ferguson, 2017; Yao and Zhang, 2018). This finding substantiates the tenets of emotional arousal theory. However, this particular exploration did not consider the impact of vocal music, replete with diverse acoustic and semantic features, on creativity. This is a significant aspect to note, as in a separate investigation using a remote-association task to measure creativity–a stark contrast from the alternative-use task – the influence of different emotional valences and emotional arousal on creative performance with instrumental music, music with lyrics, and no-music conditions produced conflicting results. The study tasked participants with producing an uncommonly utilized word that could form a logical word combination with each of the three unrelated words provided. The results indicated that background music, both instrumental and acoustic, did not foster creativity in a remote association task, instead impeding the ability to generate remote associations, whether acoustically or semantically (Threadgold, 2017). While these findings offer significant contributions to our understanding of the complex intersection between background music and creativity, they do not adequately clarify whether the observed attenuation of creativity is attributable to “acoustic interference,” “semantic interference,” or a combination of both. Further in-depth analysis of this issue therefore remains a focal point for future studies, offering an intriguing direction for subsequent research.

### The effect of musical emotional valence on creativity

Musical emotional valence is the degree to which music is emotionally pleasurable. Emotion serves as a crucial determinant of creativity, yet the exploration of the relationship between various music moods and creativity remains comparatively uncharted. Moreover, there’s an insufficiency of research focusing on the systematic and cross-cultural uniformity of music as stimuli. The current body of knowledge concerning music’s mood-evoking valence on cognitive functioning can be encapsulated within three primary perspectives. Firstly, the Broaden-and-Build Theory posits that music inducing a positive mood can stimulate a corresponding emotional state in a listener (Fredrickson, 2001). Under these conditions, listeners can expand their cognitive processes more effectively, prompting them to diverge from entrenched behavioral patterns and delve into novel, creative thought and action trajectories (Conway et al., 2013). Certain positive emotions, such as excitement, happiness, satisfaction, interest, and love, can momentarily increase cognitive processing boundaries, accelerating the recall speed of memorized information, enhancing the cognitive flexibility, and thereby fostering creativity (Fredrickson, 2004).

Contrarily, the second perspective proposes that creativity is negative mood music with somber tones. This viewpoint is substantiated by the Mind Repair Theory, which posits that those subjected to a negative mood state of mind exhibit stronger motivation to alleviate their negativity. They accomplish this through the sense of fulfillment ensuing from their creative endeavors. Further, the discordances prevalent in negative mood music introduce listeners to an expansive range of possibilities to contemplate change (Abele-Brehm, 1992; Miron-Spektor et al., 2011). At this juncture, the individual’s present mental state serves as an informational cue, harnessing negative mood to spur cognitive control for maximizing cognitive refinement and resilience. Contrary to a positive mood state, a negative one tends to reinforce comprehensive and detailed processing of existing information. It continuously prompts individuals to reassess and amend their knowledge and experiences, thereby fostering richer innovative goal concepts (Abele-Brehm, 1992). Moreover, the concept of the neutralizing effect of musical emotions stipulates that music induces feelings such as affinity, pleasure, or convergence, which are intrinsic to the rewarding experience music bestows upon its listeners (Vuoskoski et al., 2011). Certain listeners experience sorrow induced by empathetic reactions to sad sound features, learned associations, and cognitive rumination (Huron, 2011; Huron et al., 2014). Melancholic music evokes a profound esthetic emotion that pleases those with particular affective inclinations. This escalates arousal levels, thereby enhancing cognitive processing (Cassidy and MacDonald, 2010; Eerola et al., 2018; Ladinig et al., 2021; Taruffi et al., 2021).

Increasingly, research in the field of psychology is gravitating toward a third perspective concerning the dual effects of emotional valence in creativity. This perspective posits that both positive and negative emotions act as dual stimulatory pathways, each contributing uniquely to an individual’s creative capacity. Positive and negative emotions, respectively, enhance the fluency and originality of creative thought by influencing disparate facets of cognitive processing. Positive emotions primarily bolster the fluency and originality of creative ideation by enhancing cognitive flexibility, broadening the scope of cognition, and accelerating the pace of information processing. Conversely, negative emotions bolster these same creative characteristics, not by expanding, but rather by narrowing cognitive focus. This narrowing enables the reduction of cognitive resource overconsumption, fosters the maintenance of moderate emotional arousal, enhances cognitive persistence, and encourages endurance. In doing so, negative emotions contribute toward augmenting creative fluency and originality (De Dreu et al., 2008; Pessoa and Engelmann, 2010; Pessoa et al., 2012; Hu et al., 2015). A recent study confirms this dual-path theoretical model of emotional effects on creativity (Li et al., 2013). The study showcased the impact of both positive and negative emotion-induced creativity within a visual domain. Interestingly, whereas the influence of negative emotions on creativity has been confirmed in visually stimulated contexts, it remains unverified in experimental circumstances influenced by musical emotions.

### Effects of musical preference on creativity

Musical preference, conceptualized as the differential enjoyment or liking of various music genres by an individual, is molded by an intricate interplay of factors. It is chiefly influenced by psychological dimensions, such as mood, personality traits, and emotional propensities, sociocultural elements - encompassing trends, cultural identities, and peer-related effects - as well as musicianship characteristics, for instance, rhythms, harmonies, and genre derivatives. Another essential pillar in the shaping of musical preferences is music neuroscience processing, which delves into the brain’s distinct reactions toward varying musical stimuli and the subsequent development of preferences (Rentfrow and Gosling, 2003; Juslin and Västfjäll, 2008; Vuoskoski and Eerola, 2012). These predilections surface in favoritism toward certain genres, rhythms, melodies, tempos, artists, or specific compositions within the abundant musical landscape (Schäfer et al., 2013). The manifestations of these preferences underscore their multifaceted roots and highlight the diverse variables contributing to one’s musical inclinations.

Recent investigations have underscored the significant association between musical preferences and creativity. One illuminating study found that individual creativity level bore a meaningful link with a preference for diverse and intricate music genres, such as jazz, classical, or alternative music (Chamorro-Premuzic and Furnham, 2007). The implication of this preference underscores an individual’s notable degree of experiential openness and an enhanced flexibility of thought. In another compelling research endeavor focusing on musicians, it was found that individuals who favored avant-garde genres exemplified higher levels of overall creativity (Müllensiefen et al., 2015). This correlation suggests an intriguing conjecture: individuals who eagerly challenge the norms within the musical sphere concurrently exhibit a higher degree of creativity. This may well be because the very act of challenging established norms is, in and of itself, an effigy of creativity. In light of these developments, it becomes paramount to further investigate this intriguing commonality between unique musical tastes and superior creative tendencies.

Exhaustive research underscores the compelling influence of an individual’s cultural, socio-economic, and geographical milieu on their musical tastes, with considerable sway seemingly held by peer behavior and wider societal trends. Various studies suggest that familiarity with certain musical genres and the general predilections within social circles significantly shape musical preferences (Boer et al., 2011). The traditional Chinese music culture exemplifies a phenomenon referred to as the “preference for sadness” effect (Liu et al., 2021), characterized by a unique preference for melancholic emotional experiences and conditions among individuals nurtured in an environment that extols sadness. This cultural and educational imprint raises intriguing questions: Can an affinity for sad music–whether among creators or appreciators–fuel an upsurge in creative performance when engaging with music of a particular emotional type (Li et al., 2013)? Does this emotional affinity influence the creative output? Further, numerous studies have traced the impacts of individuals’ varying musical tastes and personality types on their creativity. However, these have primarily concentrated on preferences for music semantics, structure, and complexity (Rawlings et al., 2000). The potential influence of musical emotional preferences on creativity, notably, remains uncharted terrain warranting exploration. This omission represents a lacuna in our understanding of how emotional resonance with particular forms of music might inspire or inhibit creative processes, therefore demanding further examination.

The role of music in either enhancing or inhibiting creativity presents an intriguing conundrum. While previous studies offer mixed results, we posit that these discrepancies may arise from variations in several critical aspects of these studies. These include the type of musical stimuli involved, the manipulation methods of these stimuli, the genre of background music utilized, and individual disparities among participants (Kreutz et al., 2008; Kaufman et al., 2016; Oleynick et al., 2017). An insightful study used an alternative use task to examine the effects of music’s emotional tone and arousal on creativity (Ritter and Ferguson, 2017). Participants were exposed to instrumental music as an emotional stimulus for a set duration before the experiment. They were then instructed to brainstorm unconventional uses for a specified item–a “brick” in this case–under different background music conditions notable for their mood-boosting and arousing properties. The study’s findings indicated that positive music enhances creativity. However, negative musical moods and background music seemingly failed to boost creative abilities. A separate study evaluated creativity using a remote-association task (identifying compounds for three supplied stimulus words). Participants navigated through three background condition categories–instrumental, vocal, and silent. Researchers then asked participants to complete the remote-association task. They compared brain activity between instances with subjective experience of solution and instances without a solution. This study’s conclusions painted a different picture: both positive and negative background music negatively affected creativity.

Creativity remains a multifaceted construct entailing numerous intertwined cerebral processes. With its roots embedded in cognitive flexibility (involving the right hemisphere and prefrontal cortex), the genesis, amalgamation, and reformation of ideas (housed in the frontal and parietal regions), and evaluative judgments of these ideas (predominantly occurring within the prefrontal cortex), the complexity of creativity indeed mirrors the “semantic network” model of the human brain. Such a model proposes that creativity explores the novelty of connections between seemingly unrelated concepts spread across different cerebral regions (Kenett et al., 2017). However, it is crucial to note that creativity exploration processes can yield varying outcomes. Within the realm of present research, a widely acknowledged measure of creativity is the Alternative Uses Task (AUT) method, initially proposed by Guilford in 1950. AUT emphasizes thinking creatively about potential uses of an everyday item (Guilford, 1967). Participants are tasked with listing multiple alternative uses for a common object (for instance, a paper clip) within a specified time duration. The listed uses are assessed on three sub-dimensions: fluency, flexibility, and originality, rendering an overarching score of creativity. Fluency delineates the quantity of ideas generated while performing the creative task. For example, suggesting the use of a paper clip “as a hairpin” would contribute to a score of 1. Flexibility highlights the variety of response categories. For instance, responses like “paper clip,” “brooch,” and “bookmark” are considered distinct categories. Originality, on the other hand, measures the uniqueness or quality of a given answer in relation to the current pool of responses. Each unique idea that occurs below a certain probability threshold (typically 5%) adds a point to the score. With its extensive usage in creativity assessment (Kim, 1998; Mayer, 1999), the AUT has demonstrated experimental reliability and validity, boasting correlations of 0.8 and higher (Runco, 2004; Fink et al., 2010). The AUT is thus deemed an excellent proxy for creativity (Guilford, 1966; Fink et al., 2006, 2009). Neuroscientific metrics, such as EEG, PET, and fMRI, employed in relation to AUT underscore a decisive association between the primitive (as opposed to less primitive) generation of ideas with the synchronization of alpha activity in creativity-associated regions of the brain, more specifically the right hemisphere along with frontal and parietal regions (Fink and Benedek, 2014; Schwab et al., 2014; Lloyd-Cox et al., 2022).

### Overview of the research

The present study is anchored in the dual-path model of emotional valence influencing creativity. We aim to integrate an individual’s musical preferences and the effects of two prevalent types of background music - instrumental and vocal, to explore three critical questions. First, does music augment or detract from an individual’s creative abilities? Second, does instrumental or vocal background music produce a more significant catalytic impact on creativity? Lastly, given the “preference for sadness” esthetic in traditional Chinese music culture, can both emotional valences–positive and negative, foster general creativity when moderated by musical preferences? By incorporating individual-specific factors and cultural contexts, this study seeks to elucidate the nuanced relationship between musical background, emotional valence, and creativity.

Our study advances the following hypotheses: 1. There exists a differing degree of creativity across three distinct background settings: instrumental, vocal, and silence, given the stimuli of equivalent emotional valence. We anticipate the most effective creativity enhancement in the instrumental condition, followed by the silence. The vocal condition likely demonstrates the least effect. 2. An individual’s creative level differs when exposed to stimuli possessing varying emotional valence (positive, negative). This variation is observed across three background conditions (instrumental, vocal, and silence - where silence signifies the absence of emotional stimuli). We anticipate the most elevated creative performance under negative instrumental conditions, succeeded by positive instrumental and silent environments. We conjecture that the vocal background conditions for both positive and negative stimuli will be least effective. 3. Within both the negative and positive emotional states, the instrumental music environment fosters higher creativity than the silent and vocal conditions. 4. It is postulated that Chinese music with a negative emotional tone will enhance creativity more effectively than that with a positive tone under both instrumental and vocal conditions. These hypotheses, rooted in cognitive-emotional theories, aim to extend our understanding of the interplay among music, emotion, and creativity - a nexus that remains partially understood due to the diverse findings in the extant literature. In presenting these, we enrich the discourse on the utility of music as a creative stimulant against variations in musical genre and emotional context.

## Materials and methods

### Participants

A total of 118 students–from Southwest University in China and other participating institutions, aged between 18 and 31 years (*M* = 21.95, SD = 2.83)–partook in this study. The cohort was assembled through an initial questionnaire, and was screened for the formal experiment based on a survey investigating creative tendencies (*M* = 170.92, SD = 5.30), psychological health, sleep patterns, and physical condition during the preceding week. To incorporate control variables for the study, we gathered vital information such as gender, age, major, musical preferences, and contact information from the participants prior to the commencement of the formal experiment. It’s noteworthy that each participant received standardized departmental remuneration in return for a 10-min span of their participation. Moreover, all participants were right-handed, had normal or corrected-to-normal vision, confirmed normal hearing, and were native speakers of Chinese. Informed consent was obtained from each participant ahead of data compilation, and the experiment received ethical endorsement from Southwest University in China. Prior to analysis, four out of the total 118 respondents were excluded due to their failure to comprehend the requirements of the Alternative Uses Task (AUT). For this task, participants were instructed, “List as many unrelated, distinct uses of the umbrella as possible,” with a 1-sentence explanatory note for each proposed use. However, one participant merely expressed the basic function of an umbrella, while three created literary compositions around the term ‘umbrella’. Therefore, the final count of valid participants in the study amounted to 114, encompassing 69 females and 45 males (*M* = 21.82, SD = 2.42).

### Design

The study employed a mixed-factor 3 (Background Condition: Instrumental, Vocal, Quiet) × 2 (Emotional Valence: Positive, Negative) × 4 (Creativity Measures: Fluency, Flexibility, Originality, and Overall Creativity Score according to the ODT) experimental design. In this model, creativity served as the dependent variable, assessed through four distinct dimensions–Fluency, Flexibility, Originality, and the Overall Creativity Score–all of which were derived from the alternate use tasks. The independent variables comprised of the background music condition and emotional valence. The Emotional Valence factor had two levels–Positive and Negative, while the Background Music factor incorporated Instrumental and Vocal conditions, thus making Background Condition a between-subjects variable, and Emotional Valence a within-subjects variable. It should be noted that during the study’s initial phase, participants’ musical preferences collected *via* questionnaires were not deemed part of the experimental design’s core content. Their potential impact and implications may necessitate a separate, subsequent investigation.

### Materials

#### Music stimulation

For the experiment, we curated a selection of 10 compositions drawn from classical Chinese traditional music, which have been previously adopted for research (Cowen et al., 2020; Liu et al., 2021; Wang et al., 2022). These pieces were specifically chosen due to their capacity for accurately reflecting the characteristics of diverse emotions. The selected assortment comprised distinct musical potencies (positive, negative) and types of background music (instrumental, vocal): four pieces portrayed positive moods, four negative, and two exemplified calming music. To control for potential confounding variables regarding tempo, we rigorously filtered the music stimuli to maintain a consistent average speed (120 bpm for positive music and 60 bpm for negative music). We subsequently recorded the amplitude of the chosen stimuli and utilized Adobe Audition 3.0 software to harmonize all musical stimuli’s audio levels for the experiment. Thereafter, to ensure the reliability and validity of the selected stimuli, we invited 26 university students to rate the emotional pleasure and arousal of each piece of music. Previous research supports that emotional valence, particularly sadness–a common attribute in negative mood music–can indeed induce positive emotional experiences. This paradox encapsulates the ‘preference for sadness’ effect frequently witnessed in traditional Chinese music culture. Interestingly, we noted that music evoking happiness and sadness presents moderate emotional arousal compared to music that spark extreme or insignificant emotional arousal. A central tenet of our study, corroborated by previous research, is the inverted “U” shaped relationship between emotional arousal and creativity: both too little and too much emotional arousal may hamper the creative process. Based on this, we called the five conditions applied in the current study Happy Instrumental, Happy Vocal, Sad Instrumental, Sad Vocal, and Silence (no music sensing) through relevant ratings and validations.

#### Questionnaire

In the preliminary stages preceding the formal experiment, participants furnished essential demographic data and personal music preferences via a comprehensive questionnaire. The questionnaire targeted the proclivities of the participants concerning musical pieces evoking both positive and negative affective states. In its original form, the questionnaire instructed the participants to express their musical predilections tied to various emotional spectra on a five-point Likert scale ranging from 1 (indicating distinct aversion) to 5 (indicating distinct affection), with an equivalent numerical value allocated for neutrality regarding musical choices (embracing both or remaining indifferent to either category). An illustrative query included: “On a scale of 1 to 5, please express your affinity for music characterizing the emotion ‘sadness’.” The foundation for this questionnaire can be traced back to preceding studies investigating the correlation between personality and musical preferences (Rawlings et al., 1995; Rentfrow and Gosling, 2003).

#### Self-assessment of emotional state

In this study, participants’ emotional self-assessment was conducted using the Self-Assessment Manikin (SAM) Scale (Bradley and Lang, 1994). The SAM scale requests individuals to subjectively evaluate their emotional state based on a spectrum extending from profound unhappiness to euphoria, prior to and following exposure to emotionally evocative music experiences. Ratings are ascertained on a nine-point scale, with “1″ denoting the lowest level of emotional arousal and pleasure, “5″ marking a moderate level, and “9″ indicating the peak of emotional arousal and pleasure. The use of the SAM scale allowed for an integrated measure of individuals’ emotional transformations incited by various musical experiences, facilitating a richer understanding of the intimate link between music-driven emotional shifts and creativity.

#### Creativity measurement tool

Several scholars have proposed the AUT as a well-validated tool (Wilson et al., 1953), which has undergone several refinements (Fink et al., 2006). This assessment captures behavioral experimental data, thereby offering an empirical measurement of creativity. The AUT operationalizes creativity by challenging participants to envisage as many unconventional uses as possible for a randomly presented object. Endorsed by decades of psychological research, the AUT is acclaimed for its efficacy in evaluating participants’ inventive potential and creative acumen (Torrance, 1971). In alignment with current scholarly literature, the object names selected for display to the participants encompassed a book, an umbrella, and a paper clip (Fink et al., 2010; Lloyd-Cox et al., 2022). The book served as the focal object for the preliminary practice experiment, while the umbrella and paper clip were reserved for the formal experiment. In effect, the AUT encapsulates four creativity indicators: fluency, flexibility, originality, and an overall test score. Each function as familiar benchmarks to meticulously evaluate multifaceted aspects of creativity, contributing to a comprehensive interpretation of the results.

The fluency score–indicative of a participant’s ability to generate a multitude of ideas–was obtained by tallying the total array of unique ideas or alternative uses that the participant managed to propose for each given item. Flexibility, on the other hand, was measured based on the range of categories encapsulated within the subject’s submissions. More specifically, a participant’s flexibility score was determined by the number of distinct categories that their alternative uses for each item covered. To accommodate the heterogeneity and inherent complexity embedded within use categories, three undergraduate psychology students, serving as independent evaluators, assigned flexibility scores to each subject’s responses in the AUT. These scores were assigned based on the pre-established attributes of ‘flexibility,’ displaying a commendable internal consistency coefficient of 0.92. The three individual scores were then averaged to obtain the definitive flexibility score for a participant. The metric of originality (or novelty) was gaged by the sum of infrequent responses, typically those mentioned by fewer than 5% of subjects in general testing conditions. However, in this study, the originality score constituted the count of uses reported by fewer than three subjects. Each unique idea that appeared in the participant’s response set was assigned a score of one. Lastly, a comprehensive creative thinking score was computed by summating the individual scores of fluency, flexibility, and originality. This aggregate score provides a reasonable estimate of a participant’s overall creative aptitude.

In this study, participants engaged in an operation involving multiple dimensions of cognitive abilities, namely the AUT, measures of fluid intelligence (Gf), and evaluation of creative personality tendencies. To measure Gf, the study employed Raven’s Standard Progressive Matrices (SPM). Participants were required to identify appropriate patterns from a selection of options to fill in missing elements, thus completing a graphical matrix in a regular and coherent manner. This task consisted of 20 items, with a stipulated time limit of 10 min (Tourva et al., 2016). The study incorporated Williams (1979) scale to evaluate creative personality tendencies. The scale investigates four salient aspects representing the creative mind: risk-taking, curiosity, imagination, and challenge (Williams, 1979). The objective was to ascertain linkages, if any, between brain activities associated with creativity and intelligence, thereby extrapolating a broader facet of cognitive abilities that extend beyond mere creative performance. In processing the participants’ scores on the aforementioned measures, validated factor analysis methods were employed. The goal was to amalgamate these scores to generate a single latent factor, contributing to a comprehensive evaluation of the participants’ cognitive profiles (Frith et al., 2021). This integrated approach could yield effective insight into the multifaceted construct of human cognition and creativity.

#### Procedure

**Figure 1:**
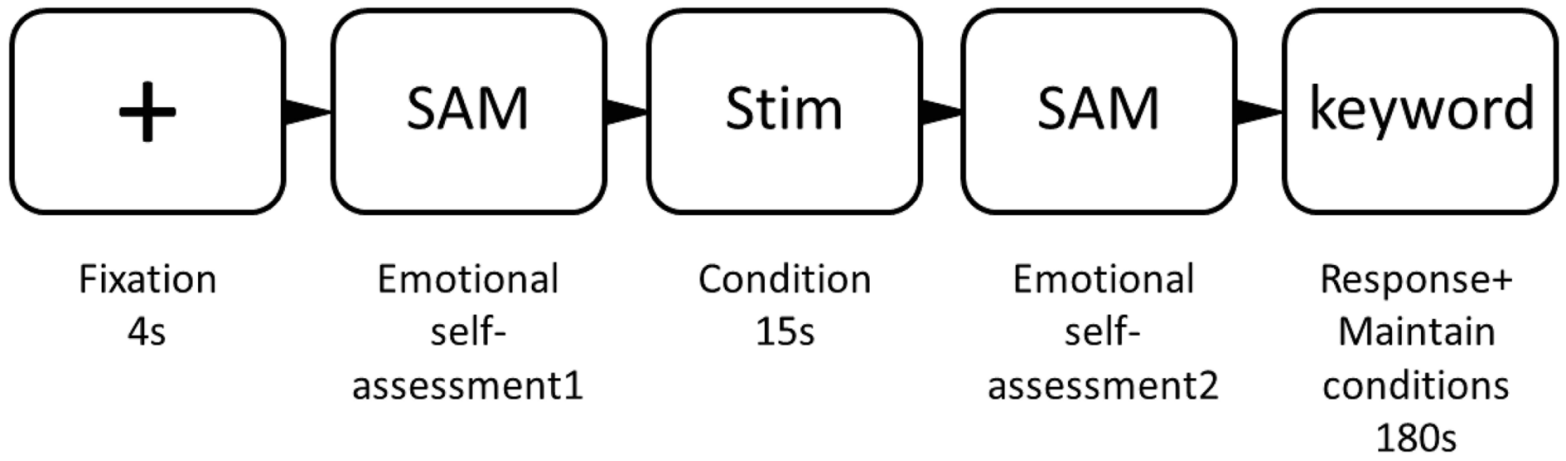
The trail procedure, from fixation (left) to response (right). Duration in seconds is presented below each frame. SAM, Self-Assessment Manikin Scale.

Participants read an information sheet, registered their information, and completed the questionnaire before participating in the experiment. The experimental task was presented *via* E-Prime 3.0 software, and participants were given instructions from AUT on how to write as many non-routine, creative-use answers as possible based on the specified item names. They could give a 1-sentence explanatory note for each answer. Before the test questions, participants were asked to solve three practice problems to ensure familiarity with the task. These practice problems were also selected (Fink et al., 2006). Participants followed the “emotional state self-assessment - different emotional musical stimuli - emotional pleasure and arousal ratings - alternative Use task” process. The experimental flow is shown in Figure 1.

The AU task commenced with the presentation of a gaze cross that lasted for a duration of 3 sec (refer to Figure 1 for the cycle of reference). Following this was the execution of the first emotional self-assessment. Subsequent to this, unambiguous music, possessing distinct acoustic variations and conforming to the characteristics of a change-state stimulus, was played through headphones for a 15-s interval. Concurrently, participants were prompted to carry out a secondary emotional evaluation, focusing on measures of pleasantness and arousal. Afterward, an on-screen keyword – representing an everyday use object – was shown to participants. They were then provided a three-minute time frame to generate inventive and original ideas correlating to the label of the randomly showcased AUT item (the idea generation phase). Simultaneously, the clearly distinguishable music was continuously played in the background for 3 min, creating an auditory backdrop during the participants’ engagement in the AU task. Participants were encouraged to attentively listen to the music while concurrently fulfilling the AU task requirements. Throughout the behavioral experiment, the randomized presentation of music corresponding to positive and negative emotional states was effectively maintained. Equally randomized was the playback of two distinct musical compositions within the same emotional category, guaranteeing that cross-emotional interference was minimized through individual three-minute intervals between any two emotion panels. In order to familiarize participants with experimental procedures and to amass valid data, a preliminary pair of short practice experiments were conducted prior to the formal experiment. During these practice experiments, the emotional states of both instrumental and vocal background music groups were moderated with calming music. Upon completion of all creativity tasks, participants were presented with a closing experiment page, prompting them to remove their headphones. They were then thanked for their participation and compensated accordingly. This accounts for the overall procedure for the AU task.

The studies were conducted in individual sections within a uniformly illuminated laboratory environment. Each compartment maintained consistent levels of brightness to ensure a controlled environment. Upon the completion of designated questionnaires and behavioral experiments, participants were permitted to depart.

## Results

### Music preference questionnaire

Table 1 reports the mean values and standard deviations for participant variables–gender, demographic data, age, music preference, and creativity including its three sub-dimensions–categorized by conditions of emotional evocation. Before delving into the hypothesis testing, an initial ANOVAs was performed to ascertain whether demographic variables such as gender or age held sway over participants’ responses. Gender, with two levels, and age, also stratified into two levels, operated as the independent variables in this analysis. Results indicated a lack of significant differences in participant scores relative to both gender and age parameters, as the value of p exceeded the 0.05 threshold, thereby deeming the effects statistically insignificant. Such a finding underscores the universality of effects across these demographic classifications, allowing for a more generalized interpretation of subsequent result analysis related to the research hypotheses.

**Table 1:** Participants’ demographic information, self-reports, and creativity quiz results.

Variable			IMG (*M*+SD) *n* = 38			VMG (*M*+SD) *n* = 39			CG (*M*+SD) *n* = 37	
Age			22.08 (1.97)			21.13 (1.66)			22.16 (1.96)	
Sex			Male = 13, Female = 25			Male = 16, Female = 23			Male = 12, Female = 25	
SAM	EV**		PP:6.57 (1.26); NP:3.41(1.46)			PP:6.92 (1.12); NP:2.41 (1.12)				
	EA**		PA:6.08 (1.34); NA:4.62(1.38)			PA:6.95 (1.31); NA:5.95 (1.41)				
Creativity levels		HP	SP	NP	HP	SP	NP	HP	SP	NP
MPQ	Creativity*	15.16(5.58)	19.08(7.30)	16.50(5.75)	12.80(3.51)	14.38(7.50)	13.00(5.43)	12.91(6.40)	17.20(5.07)	13.13(4.22)
	Fluency*	8.32(3.11)	10.08(3.52)	8.33(3.01)	6.27(1.87)	7.75(4.46)	6.94(2.38)	7.00(4.31)	9.70(3.20)	6.56(2.25)
	Flexibility	5.42(1.57)	6.77(2.42)	6.50(1.64)	5.00(1.36)	5.38(1.92)	4.81(1.68)	4.64(1.80)	5.60(2.01)	5.63(1.78)
	Originality	1.53(1.17)	2.00(0.91)	1.67(1.21)	1.27(0.80)	1.38(1.06)	1.25(0.93)	1.09(1.04)	1.80(0.92)	1.13(0.89)
Creativity levels		HM		SM		HM		SM		Quiet
AUT	Creativity**	16.71(6.23)		15.66(6.02)		13.21(5.28)		11.90(3.40)		14.16(5.36)
	Fluency*	8.92(3.20)		7.18(2.93)		6.85(2.80)		5.97(2.12)		7.54(3.41)
	Flexibility**	6.05(1.94)		5.66(1.74)		5.00(1.62)		4.64(1.11)		5.32(1.86)
	Originality**	1.71(1.10)		2.66(1.21)		1.28(0.90)		1.31(0.94)		1.30(0.97)

IMG, Instrumental Music Group; VMG, Vocal Music Group; CG, Control Group; SAM, Self-Assessment Manikin Scale;EV, Emotional Valence; EA, Emotional Arousal; PP, Positive pleasantness; NP, Negative pleasure; PA, Positive arousal; NA, Negative arousal; AUT, Alternative Use Tasks; ODT, Overall level of divergent thinking; MPQ, Music Preference Questionnaire; HP, Happy Preference; SP, Sadness preference; NP, No preference; HM, happy music; SM, sad music; *p < 0.05, ** *p* <0.01.

This study set out to investigate the influence of individual preference scores for varying musical emotions on creativity. Three distinct categories of musical emotion preferences were defined: preference for happiness-inclined music, preference for sadness-inclined music, and a neutral preference. A comprehensive analysis revealed a significant influence of musical preference on creativity, *F*(2,77) = 4.473, *p* < 0.05, $\eta_{p}^{2}=0.075$ . More precisely, individuals who indicated a propensity toward sad music demonstrated a significantly higher creative performance (*M* = 17.26, SD = 6.77) in comparison to those who favored happy music (*M* = 13.82, SD = 5.22), t_(74)_ = 2.466, *p* < 0.05, and individuals who displayed no pronounced preference (*M* = 13.61, SD = 5.03), *t*_(67)_ = 2.537, *p* < 0.05. In essence, the outcome of this study underscores the significant role of musical preferences, specifically the partiality toward sad music, as a potent contributor to creativity. These findings support the hypothesis that specific emotional dimensions of music preference can indeed have a notable effect on creative output.

In analyzing the correlations among Gf behavioral measures, creative personality tendencies, and AUT creativity, substantial evidence was found to substantiate meaningful relationships among these variables. Fluid intelligence demonstrated a significant correlation with AUT creativity, exhibiting a correlation coefficient (r) of 0.211 at a significance level of *p* < 0.01. Additionally, within the domain of creative personality tendencies, two factors, namely curiosity and imagination, were discernibly linked to creativity. Curiosity presented a correlation coefficient of 0.188, and imagination, a correlation coefficient of 0.205, both highly significant at the *p* < 0.01 level. These correlations underscore the consequential relationships between these dimensions, providing valuable insights into the intricate processes of creativity and cognitive abilities.

### Emotional arousal

**Figure 2:**
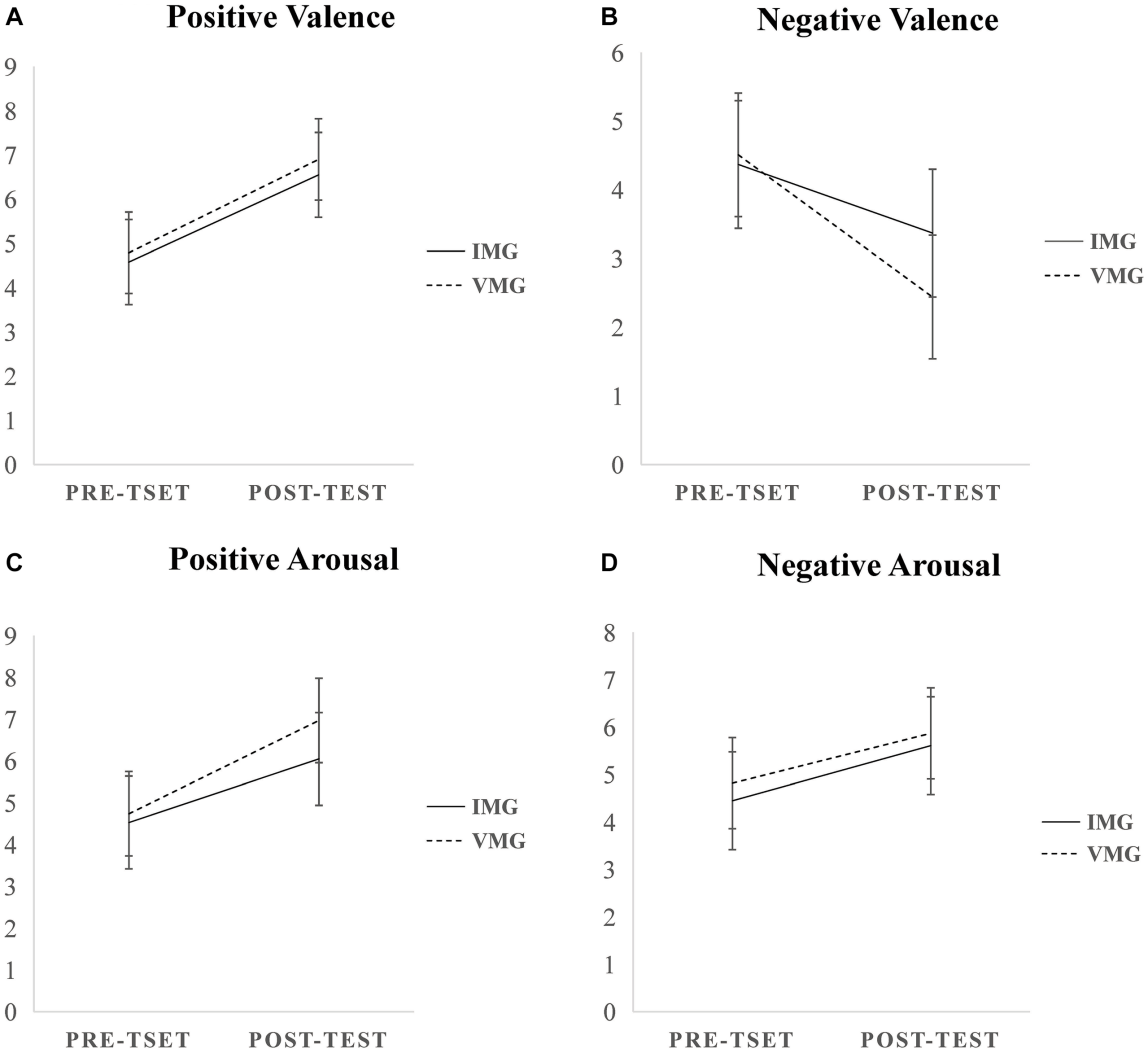
Changes in self-ratings of emotional valence and emotional arousal before and after performing the creative task with two background music types and two emotional valences. IMG, instrumental background group; VMG, vocal background group.

Figure 2 illustrates the alterations in emotional states instigated by two types of background music: instrumental and vocal - pre and post undertaking the creative task quiz. A subsequent ANOVA unveiled an interaction effect between the type of background music (instrumental, vocal) and the time point (pre-test, post-test) concerning arousal and valence. In terms of the positive emotional valence, there was a significant main effect of the time point, *F*(1,77) = 175.790，*p*<0.001, $\eta_{p}^{2}=0.540$ . Conversely, the main effect of the type of background music was not statistically significant, *F*(1,77) = 3.694, *p* > 0.05, Additionally, the interactive effect between the type of background music and the time point did not reach statistical significance, *F*(1,77) = 0.258, *p* > 0.05. For positive emotional arousal, the main effect of the time point was significant, *F*(1,77) = 117.593, *p*<0.001, $\eta_{p}^{2}=0.439$ . Furthermore, the main effect of the type of background music demonstrated significance, *F*(1,77) = 11.473, *p*<0.01, $\eta_{p}^{2}=0.071$ . The interaction effect of the type of background music and the time point also emerged as significant, *F*(1,77) = 4.512, *p*<0.05, $\eta_{p}^{2}=0.029$ . Thus, the evidence suggests that the time and type of background music interacted, influencing positive emotional arousal to a measurable extent.

Regarding the negative emotional valence, there was a significant main effect both related to time point, *F*(1,77) = 123.101, *p*<0.001, $\eta_{p}^{2}=0.451$ , and background music type, *F*(1,77) = 8.076, *p*<0.01, $\eta_{p}^{2}=0.051$ . More intriguing was the interaction effect observed between the type of background music played and the time point, which was significantly notable, *F*(1,77) = 15.080, *p*<0.001, $\eta_{p}^{2}=0.091$ . On the aspect of negative emotional arousal, there was a significant main effect of the time point, *F*(1,77) = 48.324, *p*<0.001, $\eta_{p}^{2}=0.244$ . However, the main effect of the type of background music was not statistically significant, *F*(1,77) = 3.648, *p* > 0.05. The interaction effect between the type of background music and the time point also did not yield significant findings, *F*(1,77) = 0.063, *p* > 0.05.

Subsequent ANOVAs conducted for emotional arousal and valence revealed significant variations between the two time points and the types of background music. This lends credence to the success of our mood induction procedure. Table 2 presents the mean and standard deviation of the emotional potency and emotional arousal scores and classifier performance for the two background conditions and two-time points. Changes in negative emotional pleasure and valence demonstrated substantial statistical significance for both instrumental and vocal background genres, signifying that instrumental music yielded significantly enhanced pleasure in a positive emotional context, *F*(1,77) = 22.614, *p*<0.001, $\eta_{p}^{2}=0.131$ . On the contrary, when analyzing shifts in positive emotional pleasure, valence variances between instrumental and vocal music genres were not statistically significant. This suggests that both forms of background music elicited similar pleasure levels within a positive emotional context, *F*(1,77) = 2.952, *p* > 0.05. Attention then shifted to alterations in arousal scores within a positive emotional setting, which revealed a significant increase associated with both instrumental and vocal music categories, *F*(1,77) = 15.187, *p*<0.001, $\eta_{p}^{2}=0.092$ . In comparison, alterations in arousal scores pertaining to negative emotional states evoked no statistically significant results, indicating comparable arousal responses from instrumental and vocal music during negative emotional episodes, *F*(1,77) = 2.337, *p* > 0.05.

**Table 2:** Mean and standard deviation of emotional potency and emotional arousal scores, as well as classifier performance across two background conditions and two time points.

	Instrumental music group (*n* = 38)		Vocal music group (*n* = 39)	
	Positive instrumental	Negative instrumental	Positive vocal	Negative vocal
	M ± SD	M ± SD	M ± SD	M ± SD
Pre-test emotional state				
Valence	4.579(0.92)	4.37(0.85)	4.79(0.83)	4.51(0.94)
Arousal	4.53(1.22)	4.45(1.11)	4.74(1.09)	4.80(1.03)
Post-test emotional state				
Valence	6.53(1.01)	3.37(1.02)	6.9(1.02)	2.44(0.91)
Arousal	6.03(1.00)	5.61(0.95)	6.97(0.93)	5.87(0.89)

### Creative tasks

Creativity, commonly denoted by the generation of novel ideas and perspectives, lies at the heart of this study. Utilizing the AUT, we sought to delve into its intricate workings. We proceeded to administer a three-way ANOVAs wherein the dependent variables were the collective scores of fluency, flexibility, originality, and creativity, as derived from the AUT results. The independent variables, on the other hand, were stipulated as background condition and affective valence.

**Figure 3:**
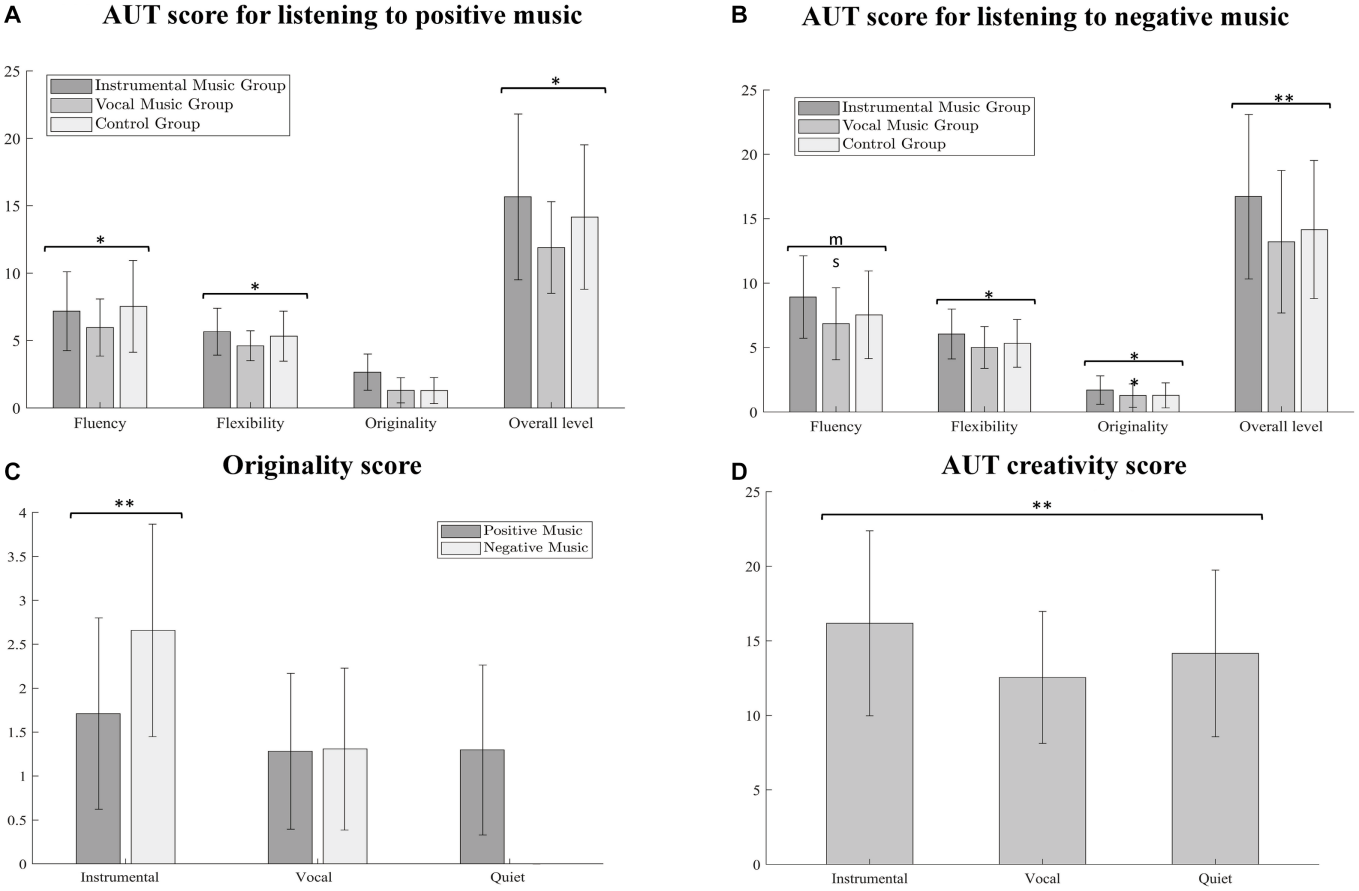
Differences in the overall levels of fluency, flexibility, originality and divergent thinking presented by the three groups of participants performing the alternative use task (AUT) under the influence of the two emotional levels. ms, significant marginal differences; *p < 0.05, **p < 0.01.

The results we obtained were quite enlightening. Notably, the influence of background condition was observed to be significant on the aggregate level of fluency, flexibility, and originality dimensions, extending to overall creativity. Interestingly, the influence of affective valence achieved statistical significance solely in relation to the fluency and originality dimensions. Furthermore, a significant interaction between background condition and affective valence was evident within the boundary of the originality dimensions. Visualization of these correlations is duly provided in Figure 3, contributing to an easier assimilation of these intricate relationships between creativity, background conditions, and affective valence.

Table 3 delineates the impact of contextual conditions and emotional validity on creativity, its three constituent dimensions, and corresponding interaction effect values. Further qualitative analysis indicated that the background condition significantly influences overarching creativity outcomes. Total creativity scores were markedly higher under instrumental music background conditions (*M* = 16.18, SD = 6.20), a stark contrast to conditions under vocal music (*M* = 12.55, SD = 4.42), t_(152)_ = 4.192, *p* < 0.001. However, no significant difference was observed when compared with conditions devoid of music (*M* = 14.16, SD = 5.59), p > 0.05. Considering creativity’s subdimension of fluency, scores were significantly escalated during instrumental (*M* = 8.05, SD = 3.20) compared to vocal (*M* = 6.41, SD = 2.49) conditions, *t*_(152)_ = 3.555, *p* < 0.01. Still, no marked discrepancy was noted between instrumental and no music (*M* = 7.54, SD = 3.41), p > 0.05. This implies that subjects demonstrated superior fluency in an instrumental music context. Assessing the subdimension of flexibility, instrumental (*M* = 5.86, SD = 1.86) and no music (*M* = 5.32, SD = 1.86) conditions rendered significantly superior flexibility compared to conditions with vocal (*M* = 4.81, SD = 1.38), t_(152)_ = 3.979, *p* < 0.001. Nevertheless, the differential between instrumental music and no music conditions was insignificant, *p* > 0.05, suggesting superior flexibility outcomes under instrumental and no-music circumstances. Turning to the subdimension of originality, scores were profoundly higher in the instrumental (*M* = 2.18, SD = 1.30) condition compared to no music (*M* = 1.30, SD = 0.97), t_(152)_ = 3.584, *p* < 0.01. Furthermore, originality scores were significantly higher in the vocal (*M* = 1.29, SD = 0.90) compared to the no music (M = 1.30, SD = 0.97) condition, *t*_(152)_ = 4.839, *p* < 0.001. These findings suggest a superlative originality performance under the influence of instrumental background music.

**Table 3:** Effect values of background conditioning and emotional valence on creativity and its three sub-dimensions.

(A) Creativity			
	*F*	*p*	𝜂𝑝2
Background Conditions	17.779	0.000	0.106
Emotional Valence	1.876	0.173	0.012
Interaction Effect	0.022	0.883	0.000

(B) Fluency			
	*F*	*p*	𝜂𝑝2
Background conditions	13.238	0.000	0.081
Emotional valence	8.349	0.004	0.053
Interaction effect	0.918	0.340	0.006

(C) Flexibility			
	*F*	*p*	𝜂𝑝2
Background conditions	15.854	0.000	0.096
Emotional valence	2.194	0.141	0.014
Interaction effect	0.000	0.985	0.000

(D) Originality			
	*F*	*p*	𝜂2
Background conditions	28.441	0.000	0.159
Emotional valence	8.511	0.004	0.054
Interaction effect	7.637	0.006	0.048

Significant results were observed in the main effects of emotional valence only in relation to fluency and originality measures. Specifically, the influence of emotional valence on fluency showed participants achieved higher scores while under a positive emotional state (*M* = 7.87, SD = 3.17) compared to when they were under a negative emotional state (*M* = 6.57, SD = 2.63), t_(152)_ = 2.769, *p* < 0.01. Interestingly, on the originality scale, participants demonstrated significantly higher scores under negative emotional conditions (*M* = 1.97, SD = 1.33) than those under positive emotional conditions (*M* = 1.49, SD = 1.01), *t*_(152)_ = 2.529, *p* < 0.01. These findings suggest that a state of negative mood may potentially enhance performance on measures of originality, indicating a possibly surprising catalyst for imaginative thinking.

The analysis of the interaction between background condition and emotional valence on originality suggests that under instrumental music conditions, the originality of negative emotions significantly exceeds that of positive emotions, *p* < 0.01. Meanwhile, under vocal music conditions, there is no significant difference in the originality of the two emotions, *p* > 0.05. These findings imply that the originality performances associated with negative emotions and instrumental music background fare better.

## Discussion

The primary objective of this study is to evaluate the collective influence of three contextual conditions, two variants of emotional valence, and individual musical predilections on the progression of creativity during ideation. The endeavor aims to address lingering uncertainties regarding the extent of the contributions made by musical emotional valence and background music types to creative activities and the cognitive operations they engender. Our initial goal comprises an exploration into whether mood music with either positive or negative valence, provided they demonstrate a similar level of emotional arousal, can enhance creativity. This hypothesized effect, which aligns with the dual-path model of emotional influences on creativity, is proposed to be moderated by an individual’s personal musical preference. The study’s second objective centers around discerning potential disparities between the impacts of various background conditions on creativity. To facilitate this, we segregated the experiment into two sequential time phases, whereby we analyzed creativity under two kinds of emotional valence within three groups, with each group exposed to a unique set of background conditions. Anticipated findings from this experiment can harbor significant implications for understanding the nuanced interface between different factors such as music, emotion and creativity, and how they coalesce to shape creative thinking and ideation over time.

Our research started with several hypotheses regarding the influence of different background conditions (taking into account the type and emotional intensity of music) on creativity. We posited that creative performance would peak when exposed to instrumental background music, perform moderately well in a quiet setting, and be the least beneficial when accompanied by vocal music. Our rationale was that instrumental background music has the potential to augment creativity by eliciting positive emotions and intensifying the level of emotional arousal. In contrast, we believed that vocal background music might hinder creativity by causing cognitive resources to be disproportionately strained and over-activated. We also posited that both positive and negatively toned music stimulate creativity. Yet, the effect of negative moods on creativity was theorized to be moderated by the listener’s music preference. Along similar lines, we hypothesized that creativity will exhibit a significant correlation with both positive and negative mood music. The results of our study suggest that the presence of background music, its type, and the intensity of its emotional appeal all effect creativity in unique ways. Collectively, our findings offer fresh insights into the interplay of music, mood, and creativity, and could potentially support the dual pathway model of mood influencing creativity. These findings not only fit into the existing body of research but bridge gaps, propose new directions for inquiry, and introduce novel connections between these multifaceted domains. In the realm of creativity, attributes such as intelligence, curiosity, and imagination gain paramount importance. These cornerstone traits undergird creative tendencies, fostering an intellectual environment conducive to the exploration and origination of novel ideas. Investigations into creativity also underscore a significant correlation between these attributes, where individuals endowed with a higher degree of intelligence, curiosity, and imagination frequently exhibit heightened levels of creativity.

### The effect of instrumental and vocal background conditions on creativity

The This research forges fresh pathways in elucidating the intricate relationships between background music, emotional responses, and creativity. As previously cited, numerous studies illustrate the role of different types of background music in activating emotional and cognitive processing within task-specific contexts (Sauter and Eimer, 2010; Loui et al., 2013; Pell et al., 2015). Furthermore, background music has been shown to significantly enhance an individual’s level of cognitive processing (Day et al., 2009). This effect primarily stems from the expected induction of positive emotions and the resultant heightening of emotional arousal states—with music serving as the catalytic agent, thereby bolstering cognitive processing (Yao and Zhang, 2018; Zhang et al., 2018). In alignment with these expectations, significant disparities were identified in the AUT scores across conditions of silence, instrumental background music, and vocal background music. The overall indices of fluency, flexibility, and creative originality were notably higher in both the instrumental background music and silent conditions relative to the circumstances under which vocal background music was present. Additionally, creative ingenuity demonstrated a significant upswing in the instrumental background music setting, as compared to both the silent and vocal background music conditions.

The present study corroborates earlier findings, positing that the presence of background music might bolster creativity. This augmentation is observed to occur through the genesis of a phenomenon that tempers disfluent thinking–a sporadic, inattentive type of cognition, rather than a coherent one (Mehta et al., 2012; Mehta and Dahl, 2019). Interestingly, nature sounds exhibiting moderate arousal levels were observed to foster a more favorable environment for creativity when compared with their low-arousal counterparts. Other studies support the idea that background music optimizes participants’ creative output (Ritter and Ferguson, 2017). In their examination, the creative performance in conditions with emotionally pleasant, arousal-inducing music was juxtaposed with conditions of silence. The comparison revealed an amplified creative performance under the musical condition, relative to the quiet environment. This observation illuminates the profound effects of emotional arousal on creativity, propounding the inference that background music plays an instrumental role in catalyzing participant’s creativity. It further implies that background music holds the potential to enhance the degree of arousal, subsequently promoting cognitive processing by eliciting specific emotional responses.

Listening to instrumental background music appears to moderately elevate emotional arousal levels. This stimulation promotes flexibility in thought processes, consequently fostering creativity enhancement (Thompson, 2012). Background music with different genres, be it instrumental or vocal, each carrying distinct emotional valence, engenders unique emotionally pleasurable experiences. This variability in experiences can induce divergent impacts on creative faculties. While vocal background music was noted to heighten pleasure levels compared to instrumental music in the context of a positive mood, the difference was found to be statistically non-significant. This could be attributable to vocal music’s dual nature as a carrier of both musical and linguistic emotional cues. This multi-modal emotional information potentially enhances positive emotional experiences in positive moods, leading to an upsurge in pleasure. This process reflects the sophisticated integration of emotional information processing (Hajcak et al., 2010). Conversely, in the negative mood scenario, this interwoven processing of emotional data may result in reduced pleasantness. This is potentially brought about by reinforcing negative emotional experiences. This aligns with the results of prior investigations emphasizing the emotional processing of different musical contexts, where participants demonstrated significantly elevated pleasure levels while listening to negatively-charged instrumental music when compared to parallel vocal works (Brattico et al., 2011; Jiang et al., 2020). This observed correlation between heightened emotional pleasantness and creativity enhancement may elucidate why instrumental background music in a negative mood setting may bolster creativity, whereas vocal background music might present obstacles to creative thinking.

Nevertheless, vocal background music, characterized by a high emotional valence value, may impede fluency, flexibility, and overall creativity. As delineated in the cognitive interference theory, adverse effects on cognitive processing may transpire when an individual’s cognitive resources come under strain. Consistent with this view, recent empirical evidence confirms that songs exert a significant interference effect on cognitive processing, while instrumental tracks prove less intrusive (Alley and Greene, 2008; Shih et al., 2012). Vocal music comprises both musical and verbal information. Consequently, when engaged in a creative task while listening to a vocal piece, an individual’s creativity may be compromised. The need to comprehend both the music and linguistic elements exacerbates demands on cognitive resources and attentional allocation, potentially distracting from the task at hand. In contrast, instrumental music may present a milder challenge for cognitive resources and attentional allocation integral to creativity, thereby offering greater fluidity and flexibility than vocal music. It has been suggested that instrumental music engenders conditions more conducive to cognitive processing as compared to both silence and vocal music. This suggests that the implementation of instrumental music as a form of background auditory stimulation may potentially bolster creativity by minimizing cognitive interference and optimizing the allocation of cognitive resources (O’Hare, 2011; Jäncke et al., 2014).

The instrumental background condition demonstrated a significant edge over both the quiet and vocal background settings in proffering originality. This observation bolsters the notion of a favorable impact of emotional arousal induced by background music on creativity. A majority of participants in this experiment were habituated to a daily ambient music environment, fostering their learning and work experiences. Consequently, these participants found the instrumental backdrop invigorating, enriching their ideation process and elevating creativity (Dobbs et al., 2011). Concurrently, these results may also be ascribed to the personality dynamics of the participants. Consistent with previous findings, extroverted individuals exhibited greater tolerance toward background music and noise during cognitive tasks as compared to their introverted counterparts (Furnham and Bradley, 1997). Contrastingly, no significant disparities were detected in fluency and flexibility across the instrumental and quiet background conditions. This might be attributable to the heightened emotional arousal elicited by the instrumental backdrop, potentially infusing an element of cognitive interference, particularly for those unaccustomed to concurrent music listening and thinking. This could consequently impose detrimental implications for fluency and flexibility. In consideration of individual differences (Gingras et al., 2015; Tavani et al., 2016), an immersive exploration and control of features like semantics, tempo, rhythm, and music type within a context-specific music genre is indispensable in delineating the relationship between background music and creativity. This is crucial for elucidating the intricate dynamics tethering them (Kämpfe et al., 2011; Doyle and Furnham, 2012).

### The effect of musical emotional valence on creativity

Indeed, our findings revealed a significant surge in the AUT scores both in conditions subject to positive and negative instrumental mood music compared to conditions devoid of musical stimuli. This aligns with the notion that both positive and negative mood music foster the stimulation of participants’ creativity and that these effects are achieved through distinct pathways, resonant with the dual-pathway model of emotion’s impact on creative cognition (De Dreu et al., 2008). At odds with our initial hypothesis that negative emotional stimuli would bolster creativity, it was found that the creative task performance under negative emotional stimuli exceeded only in terms of originality when compared to positive emotional stimuli.

Consistent with previous research and contrary to our initial expectations, our findings underscore the facilitating impact of positive emotions on creativity, chiefly manifested in the sub-dimensions of fluency and flexibility. Evidently, a positive mood catalyzes an enhancement in emotional arousal, which in turn, ascend the fluency and flexibility components of an individual’s creative capacities. In tandem, individuals ensconced in a state of happiness experience a liberation of their attentional resources, thereby fostering increased interconnectedness amid cognitive components. This interconnectedness eventually culminates in a production of myriad useful elements during the associative phase of cognition (Bolte et al., 2003), thereby augmenting fluency and flexibility. These empirical insights find resonance in the Extended-Constructive Theory, which posits that individuals immersed in positive moods perceive their environment as secure and void of threats. Consequently, they precipitate the generation of non-conforming, constructive, and innovative ideation (Rowe et al., 2007; Schmidt, 2019). Such individuals, thereby, showcase heightened creativity, contributing to the accumulating evidence of positive moods’ facilitative effect on creative endeavor.

The present investigation, drawing comparisons to previous studies, reported an interesting anomaly: higher originality was observed with negative emotional stimuli as opposed to positive emotional stimuli. This is in stark contrast with previous studies where the latter elicited higher originality scores. A plausible explanation for this could be the participants’ preference for the selected negative emotional Chinese music. Rooted deeply in traditional Chinese music culture is an esthetic appreciation that resonates with the notion of “sadness as beauty.” This emotional preference potentially moderates Chinese students’ originality when responding to negative instrumental background music (Sachs et al., 2015; Liu et al., 2022), leading to enhanced creative performance under the aegis of non-stressful, negative background music. It is equally important to consider that the musical preferences of individuals are largely shaped by exposure to specific music genres and common preferences within their peer groups. The underlying factors could range from shared cultural backgrounds, prospects for identity construction, or motivations satisfying individual psychological needs (Abele-Brehm, 1992; He, 2023), thereby directly influencing their predilections toward particular music genres (Lonsdale and North, 2011). Moreover, neurobiological responses, represented through varying reward mechanisms in the brain when we listen to preferred music, offer biological substantiation to the development of musical preferences (Huron, 2011; Salimpoor et al., 2011). These experimental findings underscore the significant role of music preferences, particularly for sad music, in the enhancement of creativity. Importantly, happy music preference, albeit contributory to creativity, did not exude a significance parallel to that of sad music preference. This might be attributed to the fact that positive music bolsters creative problem-solving more through its augmentation of the participants’ emotional arousal, rather than fostering creativity primarily through music preference.

Moreover, the present study notably showcased that music imbued with negative emotional valence is capable of fostering a semblance of pleasurable experience among the participants. This assertion is evidenced by the participants’ self-rated emotional valence. It can be speculated that the negative emotions elicited by the music induced a neutralizing effect (Sachs et al., 2015). This phenomenon, in turn, provoked individuals to forge an esthetic appreciation for music that is inherently perceived as emotionally negative, ultimately culminating in the emergence of positive emotions. Sad music, in its distinct capacity, rouses an elevated variety of esthetic emotion that translates into elicited pleasure (Huron, 2011; Juslin, 2013). Engaging with such music provokes a shift in mood, which subsequently stimulates the production of a pleasure conduit. This transmutation is integral to the cultivation of an esthetic experience synonymous with a melancholic state of mind (Vuoskoski and Eerola, 2012; Huron and Vuoskoski, 2020). The morphing into a highly pleasurable emotion state potentially augments an individual’s creativity (Li et al., 2013). In addition, the cognitive resource theory becomes instrumental in explaining how background music mirroring negative moods can accentuate cognitive persistence. This enhancement permits individuals to maintain a consistent dispersal of attention and cognitive resources. Their cognitive processing becomes increasingly refined and robust (Lassiter et al., 1996), thus fostering a conducive environment for the accentuation of originality.

Individuals experiencing negative emotions often seek to redress their mental state through the sense of accomplishment derived from creative endeavors (Abele-Brehm, 1992; Salimpoor et al., 2011). Specifically, self-selected somber music can induce a sense of psychological solace. This comforting function is evidenced by the increased levels of the hormone prolactin observed when listening to such music (Van den Tol et al., 2016). The cognitive processing elicited by negative emotions effectively regulates cognitive control. It capitalizes on cognitive refinement and flexibility, enabling individuals to perpetually refresh and reassess existing knowledge and experiences, subsequently cultivating innovative ideas. Conversely, individuals pervaded with positive emotions do not necessitate such mental rectification. Thus, their mood state does not inherently stimulate creativity. This research teases apart the complex relationship between emotional states and creative processes, illustrating the versatile influence of music on human cognition. It substantiates the premise that mood-induced musical preferences can significantly shape cognitive and creative outcomes.

### The effect of musical preference on creativity

As Our investigation culminated in the conclusion that a proclivity for sad music serves as a salient contributor in mediating the effects of musical temperament on creativity. This finding lends considerable weight to the view that an individual’s creative performance is significantly correlated with their musical preference (Ziv and Keydar, 2009; Kämpfe et al., 2011). Those who harbor a partiality for melancholy music are typically propelled by the intrinsic satisfaction derived from aligning their auditory experience with their present emotional state when engaging with music imbued with negative emotionality. This cathartic experience facilitates the generation of a more diverse range of creative ideas (Salimpoor et al., 2011). Relevant neurobiological studies corroborate these findings. Intensely pleasurable experiences evoked by listening to music are concomitant with an augmentation in cerebral blood flow to neural regions implicated in the processes of reward-seeking, mood regulation, and arousal. These regions encompass the ventral striatum, midbrain, amygdala, orbital frontal cortex, and ventral medial prefrontal cortex, all of which are integral to the brain’s reward and pleasure circuitry (Blood and Zatorre, 2001). This interplay between music, mood, and creativity expands our understanding of the neurobiological underpinnings of creativity and presents implications for the utilization of music in cognitive and emotional interventions.

In harmony with findings from prior research, the argument is presented that emotional arousal serves as a crucial catalyst in the enhancement of creativity through music. This has been confirmed by several studies that a significant correlation between the intensity of aural stimuli and preference ratings (Marin et al., 2016). However, divergent from certain existing studies, our research gleaned a noticeable enhancement in originality linked uniquely to a preference for sad music–the impact of preference for upbeat music was not sufficiently conspicuous to bolster individuals’ creativity. It has been demonstrated that sad music, when tailored to personal emotional contexts and preferences, can foster an affirmative impact on the uniqueness of an individual’s thought processes (Brattico et al., 2016; Garrido, 2017; Eerola et al., 2018). This intimates that the beneficial influence of positive mood music on creativity might principally originate from the intense pleasure and arousal it incites (Day et al., 2009), rather than from a subjective affinity for the music itself. Moreover, the enhancing effect of melancholic music on originality could be attributed to a distinctive characteristic of traditional Chinese culture, a “preference for sadness” phenomenon, where a preference for sorrowful themes exerts a positive influence on creativity. Furthermore, it also suggests that the boost in originality provided by negatively-charged emotional music could be tied to the unique “preference for sadness” phenomenon found in traditional Chinese music culture. Notably here, those with a penchant for sad music derive a pleasurable experience from such melodies (Liu et al., 2021, 2022), thereby kindling creativity. This could potentially account for our study’s finding that a preference for sad music has a notable correlation with creativity, whereas the relationship between haply music preference and creativity fails to reach the same degree of significance.

In our study, we discovered an intriguing interface between instrumental background music and negative emotions, particularly in relation to originality. Drawing from extant literature, it has been established that instrumental music not only evokes emotions, but also stimulates individual cognition. Interestingly, the music categorized as ‘negative mood’ employed within our study possessed a more tranquil rhythm and pace compared to its ‘positive mood’ counterpart. The gentle rhythm and tempo of negative mood music potentially facilitates a state of relaxation for the listeners, fostering an environment conducive to the enhancement of thought processes (Geist and Geist, 2008; Suda et al., 2008). This soothing musical climate seemingly serves as a catalyst, refining individual thinking sophistication. This enhancement, in turn, enables individuals to tap into and allocate cognitive processing resources toward deeper, more profound contemplation, hence, fostering an improvement in originality (Hirt et al., 1997; Bateson and Martin, 2013). Thus, the intricate interplay of instrumental music and negative emotion reveals the undiscovered potential of melding auditory stimuli with cognitive processes for the enhancement of creative originality.

The available evidence corroborates our prior hypotheses suggesting commonalities between traditional Chinese music and Western classical music. Our primary proposal centered around the idea that instrumental background conditions engender individual creativity (Ritter and Ferguson, 2017; Yao and Zhang, 2018), whereas conditions featuring vocal accompaniment tend to serve as a barrier. Our second conjecture rests on the belief that music conveying negative emotions can amplify individual creativity, particularly through the enhancement of imaginative prowess (De Dreu et al., 2008; Li et al., 2013). However, the influence of such music may undergo modulation by one’s personal music preferences (North and Hargreaves, 1995; Ziv and Keydar, 2009).

The conclusions drawn from our research challenge the prevailing notion suggesting that background music (Jones and Tremblay, 2000) and negative emotions (Ritter and Ferguson, 2017) obstruct creative performance. Contrastingly, our findings lend support to the hypothesis that instrumental background music exerts a bolstering effect on creativity (Schellenberg, 2005; Beaman and Holt, 2007). Specifically, our results revealed an enhancement in creative performance during the AUT when instrumental music was presented in the background. Conversely, the employment of vocal music in the background was found to compromise AUT performance. Importantly, our study offers a substantial critique of the widely accepted perspective that positive music invariably amplifies creativity. Instead, the data gathered suggests that creativity may see an uptick under the influence of negative emotional music, likely through the operation of preference effects. It appears that the role of negative emotional music in bolstering creativity, particularly in the ingenuity component of AUT, surpasses that of positive emotional music. These findings illuminate new perspectives on the intricate interplay of music, emotion, and creativity, challenging existing paradigms and encouraging further exploration of these relationships.

### Limitations and directions for future research

The current investigation is not without its limitations. Firstly, the sample size of the behavioral experiment is relatively small, which potentially compromises the generalizability of the findings. Subsequent research should consider expanding the sample size and conducting longitudinal studies to more thoroughly investigate the effects of music on creativity. Secondly, this study is predicated on the “preference-for-sadness” phenomenon observed within the cultural context of traditional Chinese music. Future research ought to explore whether the effects of music-induced mood on creativity variate between cultural musical contexts, such as traditional Chinese music and Western classical music. Thirdly, more work is needed to understand the neural processes by which background music improves creativity. After all, music is a complex variable, and there is still relatively little research on the effects of music on creativity. With future developments in neuroscience, more complex changes in brain activity for individuals to generate creative ideas while listening to background music will be presented, and more life-based creativity tasks will challenge the current research and the conclusions drawn from applying different neurophysiological measures (Deliège and Wiggins, 2006). Additionally, the Music Preference Questionnaire utilized for the study did not account for participants’ familiarity with particular genres of music. Given that music can modulate emotional engagement and cognitive resonance—thereby fashioning a personalized context that may facilitate or inhibit the creative process—the Music Preference Questionnaire assumes significance as a potentially influential variable within cognitive or affective realms. Lastly, we should direct ample attention toward individual’s cognitive states and emotional fluctuations transpiring pre, mid, and post-creative tasks. This would offer invaluable insights into the dynamic interplay of factors impacting creativity generation and progression and could serve as an avenue for further research to understand the intricate mechanisms underpinning creativity.

To deepen our understanding of the nexus between music, emotion, and cognitive processing capacities, additional research endeavors are crucial. Future investigations should keenly consider the use of advanced neurological assessments such as event-related potentials (ERPs) or functional magnetic resonance imaging (fMRI). These tools permit a more comprehensive exploration of neural markers and cerebral activity, thereby unlocking further insights into the nexus between musical stimulation and creativity. Moreover, subsequent research ought to explore the impacts of individual musical preferences and variegated personality traits on creative performance within the context of short-term musical stimuli. Such exploration provides not only behavioral evidence, but also vital neurophysiological data that might illuminate hitherto unknown facets of the topic. Altogether, the convergence of these enduring research pursuits will contribute to our broad understanding of the intricate interplay between music and cognitive creativity. These discoveries stand to drive the frontiers of our knowledge concerning human cognition, advance the scope of applicable research and potentially result in new strategies for leveraging the potent effects of musical stimulation on creative cognition.

## Conclusion

In a breakthrough investigation, this study ventures into the largely unexplored territory of the “sadness preference” effect as hypothesized within traditional Chinese music culture, and its potential influence on the enhancement of creativity, specifically incited by negative emotional music. Our findings illuminate the multifaceted relationships between the type of background music, its emotional valence, and the individual listener’s musical preference - all of which significantly impact creativity. This research furnishes fresh evidence, underpinning the intricate bond between music, emotion, and creative cognition. Remarkably, we noted that instrumental music of moderate arousal, when used as background stimulus, championed the individuals’ creative prowess. Moreover, this study affirms that melancholic music optimally nurtured individuals’ originality in performance. We, thus, posit that participants can capably tackle creative tasks when enveloped by a backdrop of either a silent environment or negative instrumental music. It is crucial to recognize that the role of music - as a promoter or an inhibitor of creativity - is distinctly governed by the particular type of background music in play. In the realm of instrumental music, the emotional arousal incited in the listener typically stimulates cognitive activation and liberates cognitive capabilities, fueling the flames of creativity. However, it is pertinent to note that in some dimensions, its influence is indistinguishable from that of a silent setting. Alternatively, when acoustic music forms the sonic backdrop, a surplus of cognitive resources and heightened emotional arousal might, paradoxically, serve to besiege the fortress of creativity. In terms of emotional valence, both positive and negative emotions have been shown to enhance creativity. Notwithstanding, the booster effect attributed to negative emotions is, so far, restricted solely to the domain of originality, with its influence potentially modulated by individual variances, such as musical preferences. Consequently, the understanding unfolds that, while beneficial, the effect of music on boosting creativity exhibits clear boundaries.

## Data availability statement

The original contributions presented in the study are included in the article/Supplementary material, further inquiries can be directed to the corresponding author.

## Author contributions

All authors listed have made a substantial, direct, and intellectual contribution to the work and approved it for publication.
